# Hydrothermal Synthesis of Multifunctional Bimetallic Ag-CuO Nanohybrids and Their Antimicrobial, Antibiofilm and Antiproliferative Potential

**DOI:** 10.3390/nano12234167

**Published:** 2022-11-24

**Authors:** Hayfa Habes Almutairi, Nazish Parveen, Sajid Ali Ansari

**Affiliations:** 1 Department of Chemistry, College of Science, King Faisal University, Al Ahsa, P.O. Box 380, Hofuf 31982, Saudi Arabia; 2 Department of Physics, College of Science, King Faisal University, Al Ahsa, P.O. Box 400, Hofuf 31982, Saudi Arabia

**Keywords:** bimetallic, Ag-CuO, hydrothermal synthesis, antibacterial, antifungal, antiproliferative

## Abstract

The rapidly growing global problem of infectious pathogens acquiring resistance to conventional antibiotics is an instigating reason for researchers to continue the search for functional as well as broad-spectrum antimicrobials. Hence, we aimed in this study to synthesis silver–copper oxide (Ag-CuO) nanohybrids as a function of Ag concentration (0.05, 0.1, 0.3 and 0.5 g) via the one-step hydrothermal method. The bimetallic Ag-CuO nanohybrids Ag-C-1, Ag-C-2, Ag-C-3 and Ag-C-4 were characterized for their physico-chemical properties. The SEM results showed pleomorphic Ag-CuO crystals; however, the majority of the particles were found in spherical shape. TEM results showed that the Ag-CuO nanohybrids in formulations Ag-C-1 and Ag-C-3 were in the size range of 20–35 nm. Strong signals of Ag, Cu and O in the EDX spectra revealed that the as-synthesized nanostructures are bimetallic Ag-CuO nanohybrids. The obtained Ag-C-1, Ag-C-2, Ag-C-3 and Ag-C-4 nanohybrids have shown their MICs and MBCs against *E. coli* and *C. albicans* in the range of 4–12 mg/mL and 2–24 mg/mL, respectively. Furthermore, dose-dependent toxicity and apoptosis process stimulation in the cultured human colon cancer HCT-116 cells have proven the Ag-CuO nanohybrids as promising antiproliferative agents against mammalian cancer.

## 1. Introduction

In recent times, the acquiring of antimicrobial resistance (AMR) by pathogenic microorganisms against several antibiotics has made it necessary to search for novel antimicrobials agents. Modern antimicrobial agents can be proven as a decisive component of future antimicrobial formulations in controlling the AMR menace. Owing to unique physico-chemical characteristics, nanoscale particles of inorganic metals and metal oxides have shown enormous antimicrobial and anti-inflammatory potential in different bio-medical settings due to their extremely small size and large surface-to-volume ratio, as compared to the large particles of the bulk material. Amongst several inorganic nanoparticles (NPs), silver (Ag), gold (Au), copper (Cu), copper oxide (CuO), titanium dioxide (TiO_2_) and zinc oxide (ZnO) NPs have been investigated extensively for their antimicrobial, anti-inflammatory and anti-cancer potential as a bare surface individual, with a bio-active surface corona and in multi-metal composites forms [1,2,3,4,5,6,7]. Due to a lack of a fully unreeled antimicrobial mechanism, metallic NPs are considered widely as acting against pathogens through multiple actions, including direct surface contact killing by releasing metal ions, distant release of metal ions and intracellular uptake/penetration. Thus, it becomes quite challenging for pathogens to acquire full AMR against the nano-antimicrobial formulations.

Despite a plethora of investigations on the antimicrobial and anti-proliferative potential of metallic NPs, researchers continue to searching for the multi-functional abilities of metallic formulations based on their physico-chemical characteristics (e.g., size, shape, dispersity, surface-charge, stability and penetrability), origin (e.g., chemical, biological and physical synthesis), and composition (e.g., surface functionalization, bio-metal composites, bi/tri metal composites etc.). Ag-NPs are the classic and the most preferred target for tuning the desired characteristics that can be exploited in biological leveling, sensor technology and many other biomedical applications [8,9]. For instance, owing to their promising anti-bacterial and anti-inflammatory properties, Ag-NPs have now been integrated into commercially available wound dressings for faster wound-healing dressings, pharmaceutical preparations and medical implant coatings [10]. The following factors play a significant role in the Ag-NP-induced antimicrobial mechanism: (i) propensity to penetrate cell walls; (ii) intracellular accumulation; (iii) destruction of the cell membrane by Ag ions and free radicals; (iv) interaction with respiratory enzymes; (v) release of reactive oxygen species (ROS); and (vi) interaction with biomolecules, such as DNA, RNA and proteins [11,12].

At the other end, copper (Cu) has been recognized as a trace element in many organisms, including animals and plants. Furthermore, reports suggest that Cu-based materials were used more than 9000 years ago, in particular for the universally recognized antimicrobial activity of Cu [13]. Despite this, Cu-based nanomaterials have been less investigated as compared to Ag-NPs, despite their promising potential, as demonstrated in the literature. Particularly, copper oxide NPs (CuO-NPs) have been widely shown as antibacterial, antifungal and anticancer agents [14]. However, in general, both Ag and Cu particles at scale demonstrate promising antimicrobial effects as compared to their bulk-size counterparts [15,16], which are likely due to the high interactions between the Ag-NPs and CuO-NPs established with the pathogen’s cell membrane-associated chemical functional groups. Despite the fact that Cu-NPs are easily oxidized in air, CuO-NPs demonstrated significantly higher bactericidal potential as a result of the formation of an oxide layer [17,18]. Taken together, there now is a rapidly growing demand for Ag- and Cu-based NPs to be incorporated into several bio-medical settings and industrial products [19,20]. Recently, the scientific world has paid increasing interest to binary metal oxides because of their wide application [21,22]. Furthermore, the physicochemical features of the bimetallic nanoparticles have been shown to be significantly distinct from those of the individual metal particles [23]. For instance, the bimetallic platinum-palladium [24], gold-palladium [25], silver-nickel [26], palladium-silver [27] and platinum-nickel [28] NPs have shown enhanced catalytic, electronic, reducing and oxidation properties, respectively. The novelty of the bimetallic NPs’ characteristics and properties rely on the synergy between the two participating metals [29]. The metal oxide component in bimetallic NPs has high intrinsic ability to trigger robust reactive oxygen species (ROS) generation, which results in curbing the proliferation of cancer cells with high selectivity [30]. Precisely, the semiconductive feature of the nanoscale metal oxide allows the electron movement within different levels of energy bands, creating reactive surfaces. Thus, during this migration, electrons may react with oxygen species to induce the generation of hydroxyl radicals and super-oxides in order to augment the oxidative damage of the DNA in malignant cells [30]. The antibacterial potential of metal oxide NPs is mostly owing to the excessive generation of ROS, singlet oxygen and hydroxyl radicals, which can cause oxidation of the bacterial membrane and consequently their death. However, oxidative stress generated by ROS is often short-lived due to their short half-life. Thus, in order to overcome the drawbacks of the individual components, a different disinfection technique that relies on the synergistic interaction of Ag^+^ and ROS is intriguing and urgently required [31]. Therefore, the development of noble metal-semiconductor nanohybrids could make it possible for a light switch to activate their bactericidal capability. Although the antibacterial, antibiofilm and antifungal potential of Ag and CuO NPs is well known, there is paucity of data on the antibiofilm, anticandidal and antiproliferative activity of Ag/CuO nanocomposites. Therefore, in the present study, we have chosen the most commonly used and generally regarded as safe antibacterial agent Ag and CuO NPs as ingredients for the synthesis of bimetallic Ag-CuO nanohybrids by a simple one-step solvothermal method. Furthermore, the amount of Ag contents in the Ag-CuO nanohybrids exhibited a remarkable influence on in vitro antibacterial, antibiofilm, antifungal and anticancer activities of the Ag-CuO nanohybrids. Overall, this study has reported a simple, low-cost, comprehensive method for the synthesis of multifunctional therapeutic bimetallic nanohybrids for the clinical management of bacterial, fungal and cancer diseases. Furthermore, the synthesized Ag-CuO nanohybrids were characterized by SEM, TEM, EDX and XRD.

## 2. Methodology

### 2.1. Synthesis of the Ag-CuO Nanaohybrids

**Figure 1:**
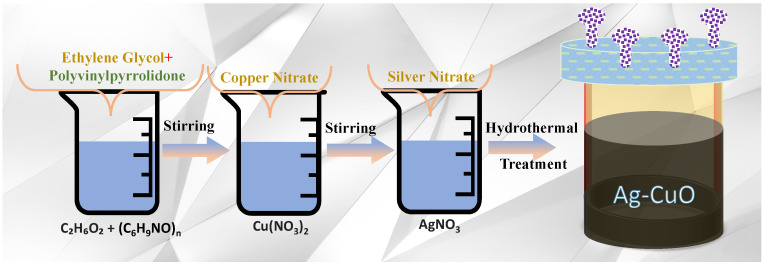
Systematic steps involved in the synthesis of the Ag-CuO nanohybrids.

Ag-CuO nanohybrids were synthesized trough the simple solvothermal method in which 0.5 g of polyvinylpyrrolidone (PVP) was dissolved in 50 mL ethylene glycol (EG) and stirred for 20 min (Figure 1). Subsequently, a copper nitrate precursor was added and further stirred for 20 min to obtain a light-blue color. A different amount of silver nitrate was added to the above homogeneous solution and further stirred for 10 min. The above homogenous mixture was further transferred to a Teflon-lined autoclave system and sealed carefully. The whole assembly was further placed inside an electric oven at 160 °C for 12 h. After completion of the reaction, the assembly was further cooled down to room temperature automatically. After being cooled down, the dark color precipitate was collated and washed with DI water and ethanol and dried for 12 h at 60 °C and used for further study. The sample was abbreviated based on the amount of silver nitrate contents. For example, Ag-CuO obtained using 0.05 g silver nitrate was abbreviated as Ag-C-1. Ag-CuO obtained using 0.1 g silver nitrate was abbreviated as Ag-C-2, Similarly, Ag-CuO obtained using 0.3 g silver nitrate was abbreviated as Ag-C-3. The Ag-CuO obtained using 0.5 g silver nitrate was abbreviated as Ag-C-4. 

### 2.2. Characterization of Ag-CuO Nanaohybrid

X-ray diffraction was performed to examine the phases of the synthesized nanohybrid in the range of 20–80°. Transmission and scanning electron microscopy (TEM and SEM) studies of the as-synthesized nanohybrid were performed to obtain the size, surface morphology and structure. Element analysis of the synthesized nanohybrids was characterized by EDX [32].

### 2.3. Evaluation of Antibacterial and Antifungal Activity

To evaluate the antibacterial and antifungal activity of synthesized nanoparticle, *E. coli* ATCC 25922 and *Candid albicans* ATCC 14053 were used. 

### 2.4. Minimal Inhibitory (MIC), Minimal Bactericidal (MBC) and Minimal Fungicidal Concentration (MFC)

Standard microbroth dilution techniques were used to examine the MIC potential of varying concentration of NPs in a 96-well microtiter plate inoculated with the tested strains. [33]. The MIC is the least amount of antimicrobial agent that can be seen to visibly inhibit 99% of bacterial growth [33]. For MBC/MFC examination, 100 μL suspensions from each well were placed over the MHA/SDA plates, respectively, and then incubated for an additional 24 h at 37 °C. The MBC/MFC value was determined by taking the lowest concentration at which there were no observable growth seen on the surface of the plates [33].

### 2.5. Evaluation of Anti-Biofilm Activity

Based on the antimicrobial results, Ag-C-1 and Ag-C-3 were selected to investigate their antibiofilm potential against *E. coli* and *C. albicans* in a polystyrene 96-well microtiter tissue culture plate, as method described by Allemailem et al. [34]. 

### 2.6. Anticancer Assay

#### 2.6.1. MTT Assay

Human colorectal carcinoma (HCT116) and human embryonic kidney cells (HEK293, all cells used in this work were purchased from American Type Culture Collection (ATCC)) were used to examine the cell viability. The 30,000 cells/well were seeded in 96-well plates containing DMEM, fetal bovine serum, L-glutamine, penicillin, streptomycin and selenium chloride and were kept in a CO_2_ incubator (Thermo-Fisher Scientific, Waltham, MA, USA) at 37 °C. The cells were then treated with varying concentration of Ag-C-1 and Ag-C-3 and further incubated for 48 h [35]. In the control group, NPs were not added. After 48 h of incubation, cells were treated with MTT (5.0 mg/mL) for 4h in the dark and then the MTT reagent was discarded, and 1% DMSO was added in each well and, finally, optical density (OD) was measured with a plate reader (Biotek Instruments, Winooski, VT, USA) at 570 nm wavelength. 

#### 2.6.2. Microscopic Analysis

The structural morphology of both the treated (5, 10, 20 μg/mL of Ag-C-1 and Ag-C-3) and untreated HCT-116 cells was observed under an inverted microscope. 

#### 2.6.3. Apoptotic Assay by DAPI Staining

The cancer cells were treated with Ag-C-1 and Ag-C-3 for 48 h in a CO_2_ incubator. After incubation, the cells were fixed with (4%) paraformaldehyde and stained with DAPI (1.0 μg/mL) under a dark environment, and then the cells were examined by using fluorescence confocal scanning microscopy (Zeiss, Jena, Germany).

## 3. Results and Discussion

### 3.1. Synthesis and Characterization of Ag-CuO Nanohybrids

**Figure 2:**
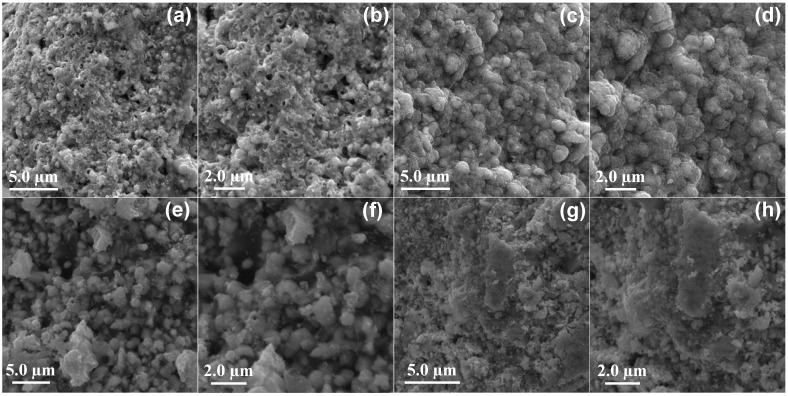
SEM images of the (**a**,**b**) Ag-C-1, (**c**,**d**) Ag-C-2, (**e**,**f**) Ag-C-3, and (**g**,**h**) Ag-C-4 nanohybrids.

The multifunctional Ag-CuO nanohybrids were synthesized as a function of the Ag concentration (0.05, 0.1, 0.3 and 0.5 g) while keeping the Cu precursors constant in a PVP (0.5 g) and EG (50 mL) reaction mixture in a Teflon-lined autoclave at 160 °C for 12 h (Figure 1). The dried powder samples of the Ag-CuO nanohybrids prepared with 0.05, 0.1, 0.3 and 0.5 g were designated as Ag-C-1, Ag-C-2, Ag-C-3 and Ag-C-4, respectively. The as-prepared Ag-CuO nanohybrids Ag-C-1, Ag-C-2, Ag-C-3 and Ag-C-4 were first examined for their morphology and shape under SEM. The SEM micrographs in Figure 2a,b have revealed that at the 0.05 g concentration AgNO_3_, the Ag-CuO (Ag-C-1) nanohybrids grew into spherical large agglomerates. Whereas, at an increased concentration of AgNO_3_, 0.1 g, as in case of Ag-C-2 synthesis, the size of the smooth-surfaced spherical Ag-CuO nanohybrids decreased substantially (Figure 2c,d). In turn, in case of the Ag-C-3 (Figure 2e,f) and Ag-C-4 (Figure 2g,h) nanohybrids, pleomorphic Ag-CuO nanostructures were obtained.

**Figure 3:**
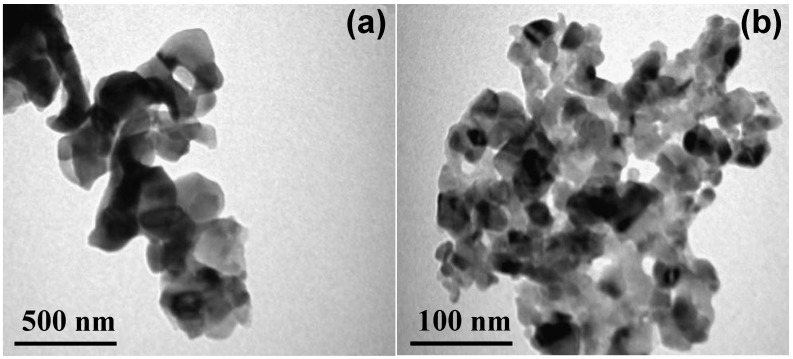
(**a**,**b**) TEM images of the Ag-C-2 nanohybrid.

The well dispersed Ag-CuO nanohybrids in sample Ag-C-2 were further analyzed with TEM at high resolution. The TEM micrographs and SAED pattern in Figure 3a,b and Figure S1 revealed that at 0.1 g concentration of AgNO_3,_ the growth of Ag-C-2 was eventually controlled within the range of 20–35 nm. The appearance of strong EDX peaks of Ag, Cu and O have confirmed that as prepared Ag-C-1, Ag-C-2, Ag-C-3 and Ag-C-4 nanohybrids do not contain impurities (Figure S2a–d, respectively) The mapping results reveled the existence of the Ag, Cu, and O in the Ag-CuO nanohybrids (Figure S3).

**Figure 4:**
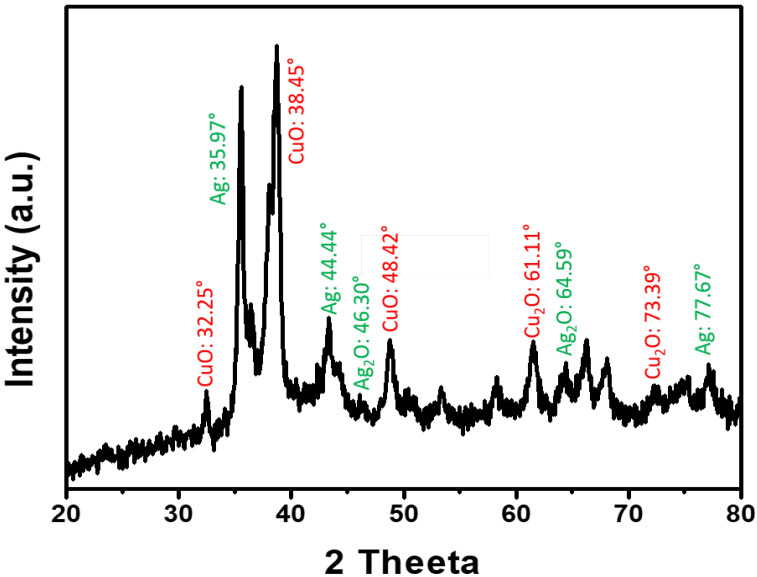
XRD pattern of the Ag-CuO nanohybrids.

The successful formation of Ag-CuO bimetallic nanohybrids was confirmed from XRD analysis as described elsewhere [36]. The XRD spectrum indicates a good crystalline structure of the Ag-CuO nanohybrids (Ag-C-2). The XRD patterns of the as-synthesized bimetallic Ag-CuO nanohybrids consist of sharp peaks, which indicated the crystallinity of the produced particles. The XRD patterns of the Ag-CuO in Figure 4 were consistent with CuO/Cu_2_O and Ag/Ag_2_O crystals. The appearance of a distinct sharp peak at 32.35°, 38.45° and 48.42° can be assigned to the (002), (111), and (202) crystal lattice of the monoclinic CuO (JCPDS 80-1268), respectively. Whereas peaks at 61.11° and 73.39° represents the (220) and (311) orientations of the Cu_2_O cubic phase (JCPDS file no 05-0667) [3]. At the other end, XRD peaks at 35.97°, 44.44°, 46.30°, 64.59° and 77.67° were assigned to the (111), (200), (220) and (311) faces of Ag (JCPDS file No. 65-2871) [37].

### 3.2. Assessment of Antimicrobial Activities of Ag-CuO Nanohybrids

#### 3.2.1. MIC and MBC Determination

**Figure 5:**
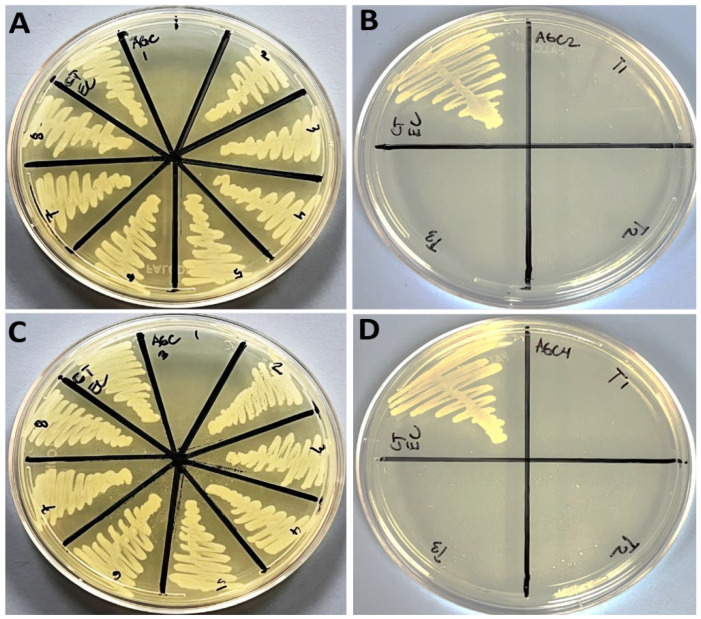
MBC (mg/mL) results of the NPs (**A**) Ag-C-1, (**B**) Ag-C-2, (**C**) Ag-C-3 and (**D**) Ag-C-4 against *E. coli*.

**Figure 6:**
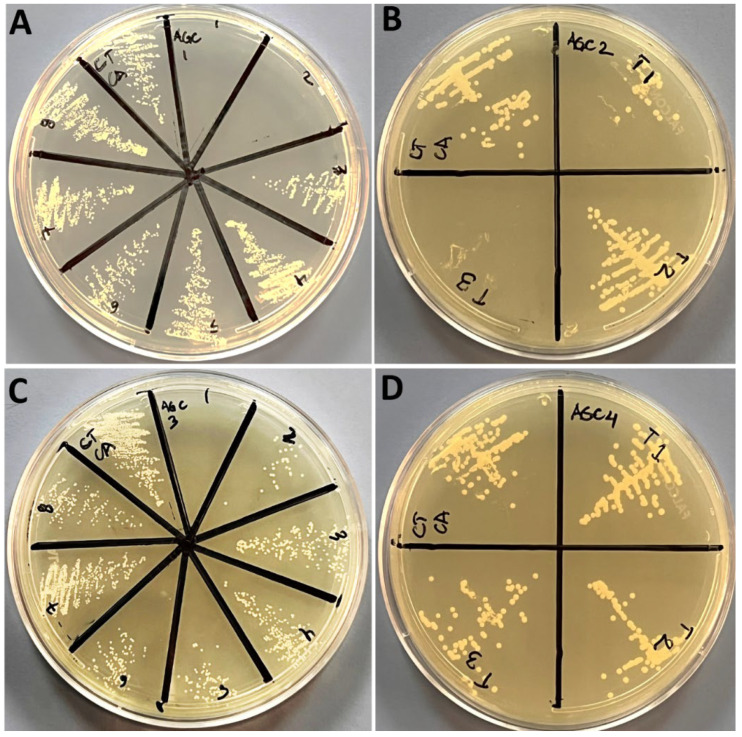
MFC (mg/mL) results of the NPs (**A**) Ag-C-1, (**B**) Ag-C-2, (**C**) Ag-C-3 and (**D**) Ag-C-4 against *C. albicans*.

Minimum inhibitory concentration (MIC) and minimum bactericidal concentration (MBC) values of all four Ag-CuO nanohybrids formulations (i.e., Ag-C-1, AG-C-2, Ag-C-3 and Ag-C-4). Data in Table 1 exhibit the MIC and MBC values of the Ag-C-1, AG-C-2, Ag-C-3 and Ag-C-4 nanohybrid formulations against the *E. coli* and *Candida albicans* strains were determined following the serial two-fold dilutions procedure, described by Ali et al. [1]. Briefly, the MIC was defined as the lowest concentration of antimicrobial agents that yielded no visible growth of the microorganisms [1]. Whereas, for MBC determination, 100 μL aliquots from the treated cell culture showing no visible bacterial growth was observed by spreading on the solid culture media plates. The MBC was considered as the endpoint that exhibits the antimicrobial agent has killed 100% of the microbial population [1]. In present study, the concentrations of Ag precursors 0.05, 0.1, 0.3 and 0.5 g in the Ag-C-1, AG-C-2, Ag-C-3 and Ag-C-4 formulations against Gram-negative *E. coli* (Figure 5A–D) and *C. albicans* (Figure 6A–D) did not exhibit concentration-dependent toxicity patterns as MICs and MBCs. In brief, the MICs and MBCs against the bacteria were observed in the range of 4 to 6 mg/mL and 8 to 12 mg/mL of the Ag-CuO formulations, whereas, under identical conditions, the MICs and MBCs against the fungal strain *C. albicans* were determined as 2 to 12 mg/mL and 4 to 24 mg/mL, and vice versa.

However, in general, bimetallic nanomaterials show synergistically high-quality antimicrobial activities against both Gram-positive *Staphylococcus aureus* and Gram-negative *Klebsiella pneumoniae* [38]. Conversely, in this study, due to the bimetallic composition, the dissolution of the bimetallic crystals and subsequent metal cations release can be speculated many-fold lesser as compared to their monometallic nanoscale counterparts. Furthermore, the differential patterns in antimicrobial activity can be argued as due to the differential (i) poly-dispersity, (ii) agglomerate formation, (iii) interaction and (iv) cell-membrane penetration propensity of the Ag-CuO formulations in culture media [39,40,41]. The activity of the bimetallic Ag-CuO nanohybrids also can be speculated due to the slow release of Ag^+^ and Cu^2+^ in the culture media. These cations, lately, bind to cell-membrane bound proteins and rapture the membrane, resulting in severe leaks in cytoplasmic fluid and leading the bacteria death [42,43]. In another study, it has been shown that Ag@CuO significantly disturbs Gram-positive and Gram-negative bacterial growth and viability as compared to CuO alone due to ROS generation and excessive damage of the bacterial cell structure [44]. In another study, the antibacterial activity of the CuO/Ag nanocomposites assessed by the disk diffusion method showed that CuO/Ag nanocomposites at concentrations of 5, 2, 1, 0.5 and 0.25 mg/mL against *E. coli* produced inhibition zone of 9, 8, 8, 7 and 6 mm, respectively [45]. They also reported that CuO/Ag nanocomposites had 98.7% efficiency against *E. coli*, whereas in this study a 100% efficiency was observed. Khashan et al. [46] reported that the CuO NPs synthesized by laser ablation method showed CuO NPs inhibit *E. coli* growth at a concentration of 1 mg/mL. Hajipour et al. [47] reported that CuO-GO-Ag nanocomposite synthesized by the chemical bath method showed MIC value of 2.6 ± 0.5 mg/mL against *E. coli*, which is parallel to our finding. In another study, it was found that the polyindole/Ag-Cuo nanocomposites prepared by the reflux condensation method showed higher activity against *E. coli* than that of CuO [48]. El-Nahhal et al. [49] showed that the functionalization of CuO-coated cotton with Ag NPs has enhanced the antibacterial performance of the fabric because of the synergistic behavior of CuO and Ag.

#### 3.2.2. Antibiofilm Activities of Ag-CuO Nanohybrids

**Figure 7:**
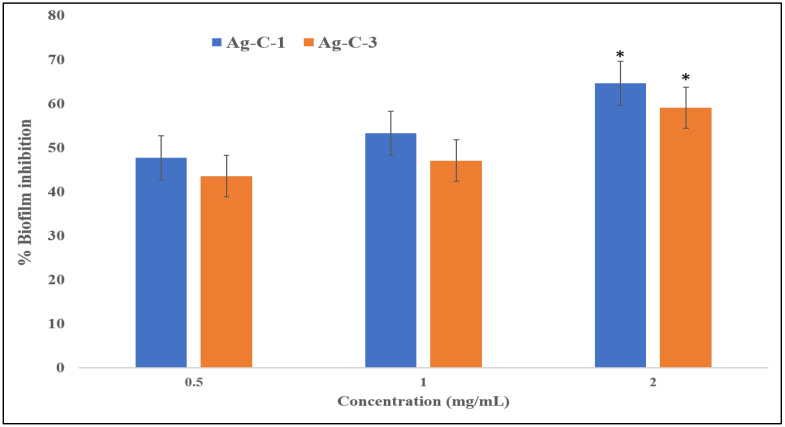
Effects of Ag-C-1 and Ag-C-3 on the biofilm-forming abilities of *E. coli*. * *p* < 0.05.

**Figure 8:**
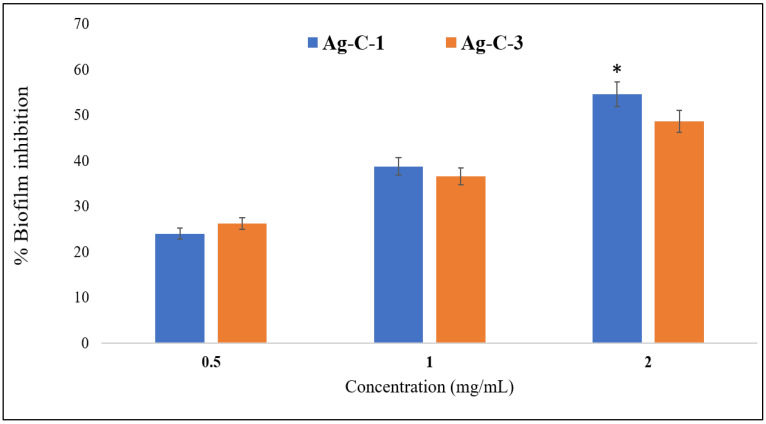
Effects of Ag-C-1 and Ag-C-3 on the biofilm-forming abilities of *C. albicans*. * *p* < 0.05.

Bacterial biofilm is a rather complex and heterogeneous matrix which contains cell aggregates/clusters of bacterial, exopolysaccharide, eDNA, eRNA, proteins, organic acids and bio-active enzymes, which ultimately protects persistent bacterial cells from antibiotic exposure and leads the AMR development [50,51]. In fact, several traditional antimicrobials, when get in contact with dense biofilms, lose their functional abilities [52]. Fortunately, metallic NPs exhibit promising antimicrobial effects on drug-resistant strains due to prolonged stability and penetrability to prevent the biofilm formation. Particularly, metal oxide NPs reflected a strong antimicrobial effect and high thermal stability while possessing effective inhibitory potential against both Gram-positive and Gram-negative bacterial biofilm as well as *Candida* fungi. In fact, most of the metal oxide NPs trigger ROS to augment oxidative stress [53,54]. Though the antibiofilm potential of Ag and CuO NPs has been shown in the literature, very little work is available in the literature regarding the antibiofilm activity of Ag/CuO nanocomposites. Therefore, in the present study, the antibiofilm potential of the Ag/CuO nanohybrid was investigated against *E. coli* and *C. albicans*. Our Ag-CuO nanohybrids have shown a dose-dependent inhibitory effect against biofilm formation of *E. coli* and *C. albicans.* The data in Figure 7 and Figure 8 demonstrate a dose-dependent (i.e., 0.5, 1.0, 2.0 mg/mL) effect of Ag-C-1 and Ag-C-3 on biofilm formation of *E. coli* and *C. albicans*, respectively. The Ag-C-1 and Ag-C-3 nanohybrid formulations, prepared with 0.05 g and 0.3 g AgNO_3_, respectively, exhibited 47.66, 53.23 and 64.59%, and 43.53, 47.05 and 49.05% inhibition in *E. coli* biofilm formation at 0.5, 1.0 and 2.0 mg/mL concentrations, respectively (Figure 7). Whereas, under identical conditions, 24.04, 38.81 and 54.67%, and 26.29, 36.69 and 48.68% inhibition in *C. albicans* biofilm was found, respectively (Figure 8).

### 3.3. Antiproliferative Activities of Ag-CuO Nanohybrids

#### 3.3.1. Cytotoxicity Analysis of Ag-CuO Nanohybrids

**Figure 9:**
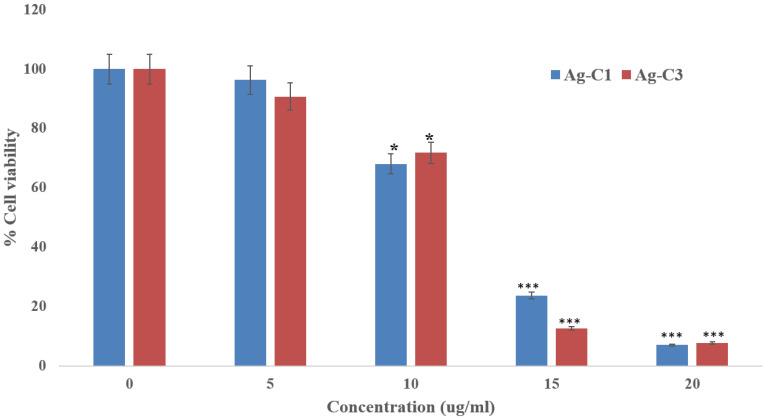
MTT assay showing percent cell viability of the HCT-116 cells after treatment at different concentration of Ag-C-1 and Ag-C-3. * *p* < 0.05, *** *p* < 0.001.

Cancer ranks as the second-leading cause of death worldwide. The International Agency for Research on Cancer (IARC) projected that there were roughly 18.07 million new cases of cancer and 9.5 million cancer-related deaths worldwide. Colorectal cancer (CRC), which includes colon and rectal cancer, is the third most common cancer in the world, following lung and breast cancer [55]. Using the as-synthesized Ag-C-1 and Ag-C-3 nanohybrids, we aimed to assess their cytotoxic potential against human colorectal carcinoma cell (HCT-116) by performing an MTT assay. Indeed, the MTT assay is employed to measure the viability of cells, treated and untreated, based on their metabolic activity through quantitating the NADPH-mediated cellular oxidoreductase enzymes, which reduce the MTT dye into insoluble formazan. The MTT assay of HCT-116 cells treated with Ag-C-1 and Ag-C-3 nanohybrids (5–20 μg/mL) exhibited significant (* *p* < 0.05, ** *p* < 0.01) cytotoxicity after 24 h. The MTT results showed a 3 ± 0.2, 32 ± 0.6, 78 ± 0.3 and 91± 0.6% decline in viability of HCT-116 cell after exposure with 5, 10, 15 and 20 μg/mL of Ag-C-1 (Figure 9). Whereas, under identical conditions, the Ag-C-3 nanohybrids induced a 9 ± 0.5, 30 ± 0.4, 87 ± 0.6 and 93 ± 0.8% decline in cell viability, respectively (Figure 8). Furthermore, the linear curve fitting of the dose-dependent toxic trends of the Ag-CuO nanohybrids yielded IC_50_ values at 11.78 µg/mL (R^2^ = 0.9336) and 11.47 µg/mL (R^2^ = 0.9162) for the Ag-C-1 and Ag-C-3 nanohybrids, respectively.

#### 3.3.2. Effects of Ag-C-1 and Ag-C-3 Nanohybrid on Morphology of HCT-116 Cells

**Figure 10:**
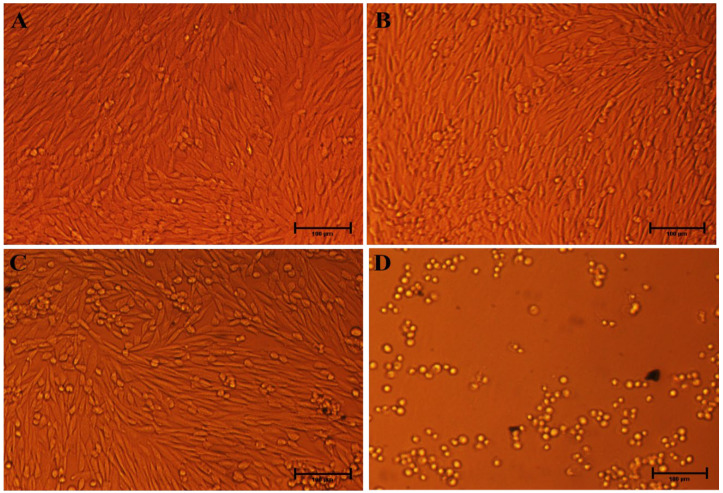
The morphological representation of the HCT-116 cells treated with different concentration of Ag-C-1. (**A**) Control HCT-116 cells; (**B**–**D**) treated with 5, 10 and 20 µg/mL. Scale bar 100 µm.

**Figure 11:**
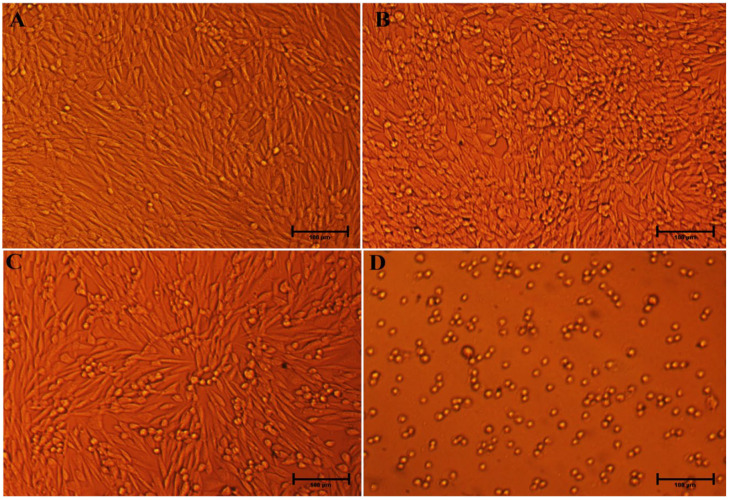
The morphological representation of the HCT-116 cells treated with different concentration of Ag-C-3 (**A**) Control HCT-116 cells; (**B**–**D**) treated with 5, 10 and 20 µg/mL. Scale bar 100 µm.

Upon nano-drugs exposure, the morphological damages in cultured mammalian cells can be clear-cut signs of the cytotoxicity of NPs, interaction of NPs with cells and internalization of NPs into the cells [32]. Similarly, we also strived here to validate our cytotoxicity outcomes by visualizing the Ag-C-1 and Ag-C-3 nanohybrids treated and untreated with HCT-116 cells. Representative microscopic images in Figure 10 and Figure 11 demonstrate the loss of native shape and size of the HCT-116 cells in treated experiments. Precisely, compared to the untreated control (Figure 10), the cells treated with 5, 10 and 20 µg/mL of Ag-C-1 nanohybrids suggest strongly the loss in plasma membrane and structural integrity, which were likely due to strong physical interaction between the Ag-C-1 nanohybrids and HCT-116 cells (Figure 11B–D) [56]. Similarly, compared to the untreated control (Figure 11A), a dose-dependent internalization of the Ag-C-3 nanohybrids into HCT-116 cells can be speculated after 24 h exposure (Figure 11B–D). A significant cytoplasmic shrinkage in HCT-116 cells evidently supported the internalization of Ag-C-3 nanohybrids through cell membrane penetration as the main cell-killing mechanism. 

#### 3.3.3. Study of Nuclear Morphology Changes of Ag-C-1 Nanohybrids Treated HCT-116 Cells

**Figure 12:**
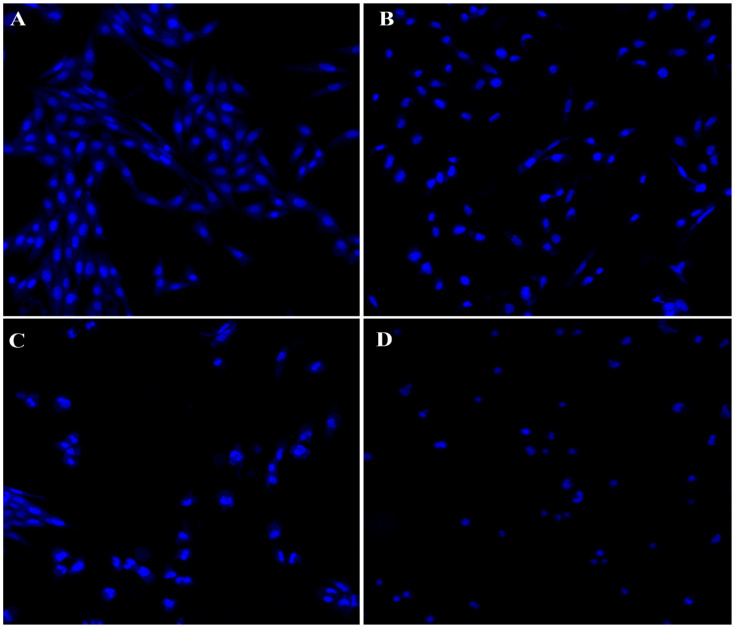
Impact of AgC1 on HCT-116 cells stained with DAPI after 48 h treatment: (**A**) control cell; (**B**–**D**) treated with 5, 10 and 20 µg/mL of Ag-C-1. Scale Bar 400 µm.

Precisely, during apoptosis stimulations, transcriptional expression apoptotic genes, such as caspase-9, caspase-3 Bax and p53, have an essential role. At the same time, nuclear DNA fragmentation is also a key sign of apoptosis induction. Therefore, we strived to evaluate our as prepared Ag-C-1 nanohybrids formulations for apoptotic activity. To study the changes in nuclear morphology in Ag-C-1 nanohybrids induce by HCT-116 cells, the cells were stained with DAPI after 24 h of exposure before microscopic examination (Figure 12). The Ag-C-1 nanohybrids induced apoptosis in HCT-116 cells was observed with a decrease in blue fluorescence intensity of nuclear DNA at the 5, 10 and 20 µg/mL Ag-CuO formulations (Figure 12B–D), as compared to the highly intense florescent control cells (Figure 10A), thereby indicating the beginning of apoptosis progression by nicking in DNA [57,58]. A recent study of Acharya et al. (2021) on biosynthesized Ag-NPs supports our Ag-C-1-induced apoptosis results strongly. Based on DAPI and flow cytometry analysis, Acharya et al. [59] have demonstrated that their Ag-NPs could perfectly induced apoptosis in Human Colon Cancer Cell HCT-116 in a dose-dependent manner.

## 4. Conclusions

In conclusion, the synthesis of Ag-CuO bimetallic nanohybrids via the one-step hydrothermal process was successfully achieved. The as-obtained Ag-CuO bimetallic nanohybrids Ag-C-1, Ag-C-2, Ag-C-3 and Ag-C-4 showed an average size in the range of 20–35 nm. TEM- and SEM-based morphological examinations of the Ag-CuO nanohybrids have ascertained that the majority of the Ag-CuO particles were spherical. Furthermore, the EDX-based elemental analysis of the Ag-C-1, Ag-C-2, Ag-C-3 and Ag-C-4 formulations has confirmed their bimetallic composition by demonstrating the presence of strong signals of Ag, Cu and O. The XRD results confirmed further their crystalline nature and bimetallic composition through characteristics miller indices and diffraction peak planes of Ag and Cu. The Ag-CuO nanohybrids have shown promising in vitro antimicrobial activities against *E. coli* and *C. albicans* strains. In addition, the cytotoxicity trends of the Ag-CuO nanohybrids against cultured HCT-116 cells are provocative enough to investigate their dose-dependent antiproliferative potential further as bimetallic anticancer nano-drugs. Overall, the results in this study are anticipated to establish various innovative directions towards bimetallic nanocomposites as potential multi-functional nano formulations in the area of nano-biomedicine and nano-biotechnology.

## Figures and Tables

**Table 1:** MIC and MBC/MFC (mg/mL) values of the NPs against the tested pathogens.

NPs	*E. coli*		*C. albicans*	
	MIC	MBC	MIC	MFC
Ag-C-1	4	8	2	4
Ag-C-2	6	12	10	20
Ag-C-3	4	8	4	8
Ag-C-4	6	12	12	24

## Data Availability

The data presented in this study are available on request from the corresponding author.

## Supplementary Materials

The following supporting information can be downloaded at: https://www.mdpi.com/article/10.3390/nano12234167/s1, Figure S1: SAED pattern; Figure S2: EDX spectra; Figure S3: Elemental mapping images.
