# Supplementary material for: Hydrothermal Synthesis of Multifunctional Bimetallic Ag-CuO Nanohybrids and Their Antimicrobial, Antibiofilm and Antiproliferative Potential
Source: Nanomaterials (Basel). 2022 Nov 24;12(23):4167. doi: 10.3390/nano12234167 (PMC9737815; doi:10.3390/nano12234167)
Supplement: Supplementary file 1 [file nanomaterials-12-04167-s001.zip › nanomaterials-2035905-supplementary.pdf]

nanomaterials

Supplementary Materials

Hydrothermal Synthesis of Multifunctional Bimetallic Ag-CuO Nanohybrids and their Antimicrobial, Antibiofilm and Antiproliferative Potential

Hayfa Habes Almutairi [1,*], Nazish Parveen [1,*] and Sajid Ali Ansari [2]

[1] Department of Chemistry, College of Science, King Faisal University, Al Ahsa, Saudi Arabia, P.O. Box 380, Hofuf 31982
[2] Department of Physics, College of Science, King Faisal University, Al Ahsa, Saudi Arabia, P.O. Box 400, Hofuf 31982
* Correspondence: halmutairi@kfu.edu.sa (H.H.A.); nislam@kfu.edu.sa (N.P.); Tel.: +966-135897537 (H.H.A)

1. Selected area electron diffraction (SAED) pattern

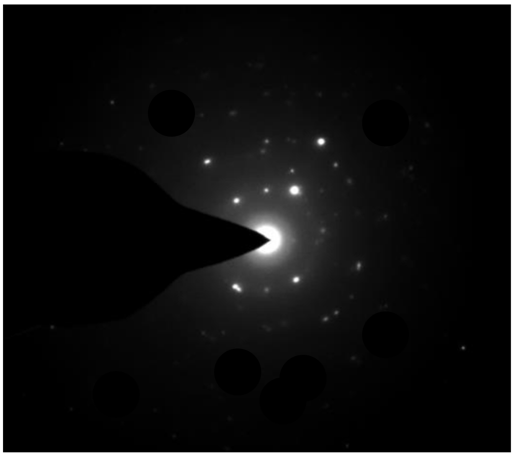

Figure S1. Selected area electron diffraction (SAED) pattern of the Ag-CuO nanaohybrid.

2. EDX analysis

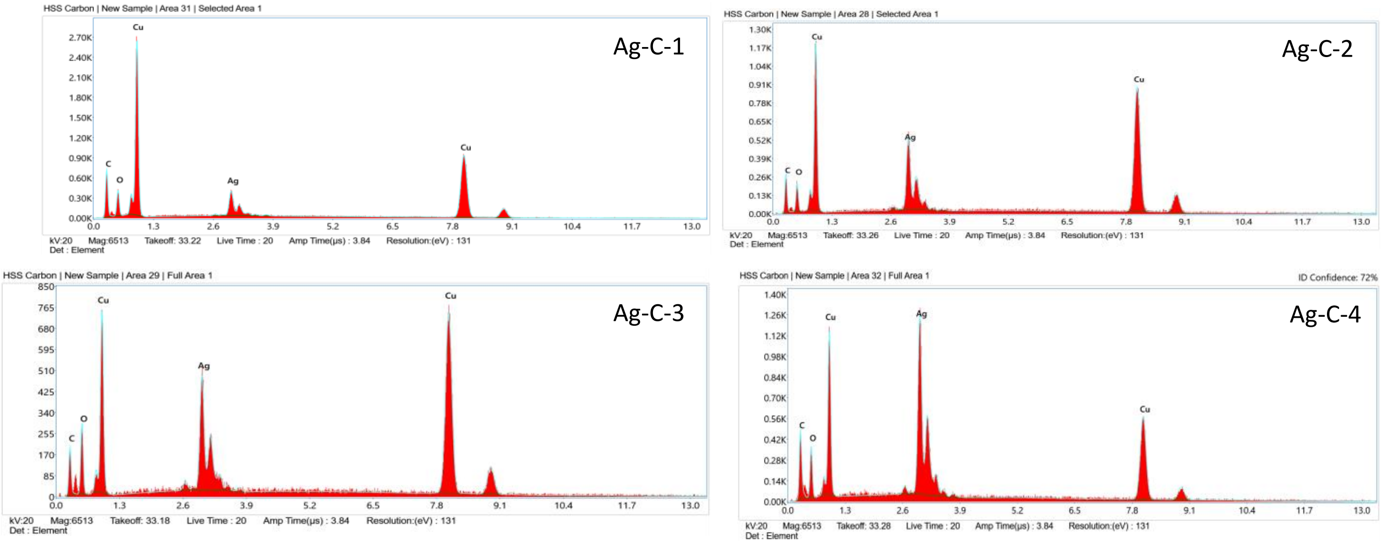

Figure S2. EDX spectra of (**a**) Ag-C-1, (**b**) Ag-C-2, (**c**) Ag-C-3, and (**d**) Ag-C-4.

3. Element mapping

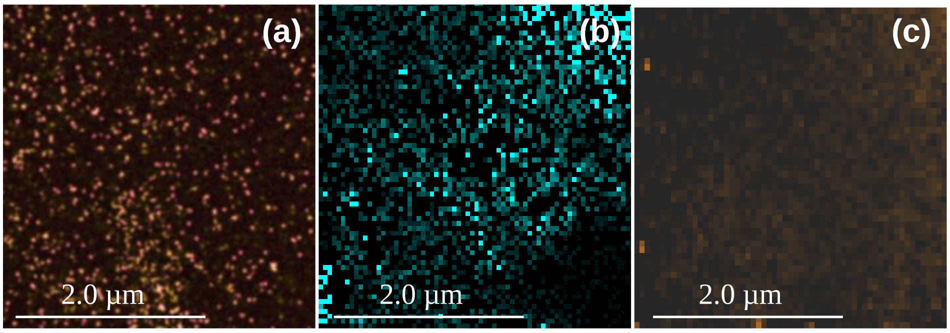

Figure S3. Element mapping of (**a**) Ag (**b**) Cu, and (**c**) O present in the Ag-CuO nanohybrids.
